# Impaired TIP60-mediated H4K16 acetylation accounts for the aberrant chromatin accumulation of 53BP1 and RAP80 in Fanconi anemia pathway-deficient cells

**DOI:** 10.1093/nar/gkv1019

**Published:** 2015-10-07

**Authors:** Emilie Renaud, Aurelia Barascu, Filippo Rosselli

**Affiliations:** 1 Univ Paris-Sud, Laboratoire «Stabilité Génétique et Oncogenèse», Equipe Labellisée La Ligue Contre Le Cancer, 94805 Villejuif, France; 2 CNRS - UMR 8200, 94805 Villejuif, France; 3 Gustave Roussy, 94805 Villejuif, France

## Abstract

To rescue collapsed replication forks cells utilize homologous recombination (HR)-mediated mechanisms to avoid the induction of gross chromosomal abnormalities that would be generated by non-homologous end joining (NHEJ). Using DNA interstrand crosslinks as a replication barrier, we investigated how the Fanconi anemia (FA) pathway promotes HR at stalled replication forks. FA pathway inactivation results in Fanconi anemia, which is associated with a predisposition to cancer. FANCD2 monoubiquitination and assembly in subnuclear foci appear to be involved in TIP60 relocalization to the chromatin to acetylates histone H4K16 and prevents the binding of 53BP1 to its docking site, H4K20Me2. Thus, FA pathway loss-of-function results in accumulation of 53BP1, RIF1 and RAP80 at damaged chromatin, which impair DNA resection at stalled replication fork-associated DNA breaks and impede HR. Consequently, DNA repair in FA cells proceeds through the NHEJ pathway, which is likely responsible for the accumulation of chromosome abnormalities. We demonstrate that the inhibition of NHEJ or deacetylase activity rescue HR in FA cells.

## INTRODUCTION

DNA interstrand crosslinks (ICLs) are DNA lesions induced by endogenous cellular metabolites, such as malondialdehyde, and by exogenous anti-cancer chemotherapeutic drugs, such as mitomycin C (MMC) and cisplatin. Bridging the opposite complementary DNA strands, ICLs prevent the opening of the double helix and represent an impassable obstacle for the progression of replication forks. A major outcome of the collision of a replication fork with an ICL is the formation of one or two one-ended double-stranded DNA breaks (DSBs). DSBs constitute the substrate for the resumption of replication and initiate homologous recombination (HR)-based mechanisms to preserve genome integrity (1–3). HR failure during DSB repair is extremely deleterious because the c-NHEJ pathway could allow cell survival at the expense of intrachromosome deletions and chromosome rearrangements.

Genomic deletions (4) and chromosome rearrangements (5,6) are the major cellular hallmarks of Fanconi anemia (FA), a rare human hereditary syndrome featuring bone marrow failure, predisposition to cancer and cellular and chromosomal hypersensitivity to ICL-inducing agents (7–10). FA arises from biallelic inactivating mutations in one of 17 identified *FANC* genes. In response to stalled replication forks, eight FANC proteins, FANCA, B, C, E, F, G, L and M, assemble into the nuclear FANCcore complex to monoubiquitinate FANCD2 and FANCI. Ub-FANCD2 and Ub-FANCI relocate to damaged chromatin, where they are a part of a large network of proteins involved in checkpoints, replication and DNA repair. The third group of FANC proteins, which includes FANCD1/BRCA2, FANCJ/BRIP1, FANCN/PALB2, FANCO/RAD51C, FANCP/SXL4, FANCQ/XPF and FANCS/BRCA1, contributes to the processes that rescue stalled replication forks and DSBs by HR (11–13). FA pathway loss-of-function impacts the cell's capability to optimally resume replication and/or re-establish genome integrity (7,10,12,14). Accordingly, the assembly of HR master proteins, such as the MRE11-RAD50-NBS1 (MRN) complex, BLM and RAD51, onto the chromatin following exposure to an ICL-inducing agent has been reported to be defective in FANCcore complex- and FANCD2-deficient cells (15–19). Importantly, although not validated in mouse models (20), it has been reported that NHEJ inhibition or downregulation can, at least partially, recover FA-associated cellular hallmarks in *C. elegans*, chicken DT40 cells and human cells (21,22). However, no previous studies have identified how the FANCcore complex or FANCD2 deficiencies result in HR impairment. Interestingly, it has been reported that the acetyltransferase TIP60 constitutively interacts with FANCD2 and that its depletion sensitizes DNA repair-proficient cells to MMC without affecting Ub-FANCD2 relocalization to subnuclear foci or the ability of FANCC-deficient cells to survive MMC exposure (23,24). Thus, TIP60 has been proposed as an integral part of the FA pathway downstream Ub-FANCD2. The role of TIP60 in guiding cells toward HR instead of NHEJ during the repair of ionizing radiation-induced DSBs has been well characterized. Indeed, the TIP60-mediated H4 acetylation of lysine 16 impedes the access of 53BP1 to nearby dimethylated histone H4 K20 (H4K20Me2) (25) and allows the resection of the DSB extremities to create 3′-overhangs, which are required for the assembly of RAD51 and the initiation of HR (26–28).

We decided to investigate why HR fails in FANCcore complex-deficient and FANCD2-deficient cells, which have a fully competent enzymatic HR machinery. We show that a) 53BP1 foci over-accumulate in the nuclei of FANCcore complex-deficient and FANCD2-deficient cells following MMC treatment; b) 53BP1 accumulation is due to the local maintenance of the chromatin in a hypoacetylated state as a consequence of the failure of TIP60 accumulation at damaged chromatin; and c) siRNA-mediated depletion of 53BP1 as well as exposure to deacetylase inhibitors significantly restores MMC resistance in FA cells by favoring the HR process. Our data suggest that a major function of the FA pathway, directly mediated by Ub-FANCD2, is to ensure that TIP60 acetylates H4K16 at the appropriate time and place to block the binding of the anti-HR and pro-NHEJ protein 53BP1 to H4K20Me2.

## MATERIALS AND METHODS

### Cell culture, treatments and transfection

Human cell lines were grown in DMEM (Gibco) media supplemented with 10% fetal calf serum (FCS) (Dutcher), 1 mM sodium pyruvate, 100 U/ml penicillin and 100 μg/ml streptomycin (all from Gibco) at 37°C in 5% CO_2_. In addition to HeLa cells, we used the following SV-40 immortalized fibroblasts: (i) PD331 cells (Fanconi anemia complementation group C fibroblasts transduced with an empty vector) and PD331-Corr cells transduced with a wild-type *FANCC*; (ii) PD20 (FANCD2 fibroblasts), PD20 Corr (a PD20-derived cell line expressing wt FANCD2) and PD20-K561R cells (a PD20-derived cell line ectopically transfected with a non-monoubiquitinable FANCD2).

Mitomycin C (MMC) (Sigma) was used for pulse (1 μg/ml/1 h) or chronic treatment (200 ng/ml). A DNA-PK inhibitor (NU7441, Tocris) and Trichostatin A (T8552, Sigma) were used at 10 μM and 0.5 μM for 2 and 5 h before chronic MMC treatment, respectively.

53BP1 and RAP80 SMARTpool siRNAs were obtained from Dharmacon. Transfection of siRNA (20 nM) was performed with Interferin (PolyPlus transfection), and the experiments were carried out 48 h later.

For clonogenic survival, 48 h after transfection, 300 cells per 6-cm plate were plated 6 h before 1 h of MMC treatment (50 or 100 ng/ml). The cells were washed twice with PBS, and fresh medium was added. Ten days later, the clones were stained with methylene blue and counted. Each point represents the mean of three different experiments.

### Western blotting

Cells were collected and disrupted in lysis buffer (50 mM Tris pH 7.5, 20 mM NaCl, 1 mM MgCl_2_, 0.1% SDS supplemented with protease and phosphatase inhibitors (Roche) and 0.1% endonuclease benzonase (Millipore)). After 10 min of incubation at room temperature, the lysates were combined with 4× Laemmli buffer containing β-2-mercaptoethanol and denatured by boiling. The proteins were separated by SDS-PAGE. The antibodies used were directed against FANCD2 (sc-20022, Santa-Cruz), actin (sc-1616, Santa-Cruz), vinculin (ab18058, Abcam), RAP80 (ab124763, Abcam), GAPDH (ab9484, Abcam), lamin B1 (ab20396, Abcam), histone H3 (ab1791, Abcam), Tip60 (sc-5725, Santa-Cruz), 53BP1 (MAB3802, Millipore), H4K16Ac (07-329, Millipore), H3K9Me3 (6F12-H4, Millipore), H3K9Ac (39585, Active Motif), H4K20Me2 (39174, Active Motif) and HA (26183, Thermo).

### Immunofluorescence

Cells grown on glass cover slips were fixed in 4% formaldehyde supplemented with 0.1% Triton X100 for 10 min at room temperature before permeabilization in 0.5% Triton X100 for 5 min. After blocking with 3% BSA in PBS containing 0.05% Tween 20, the cells were stained for 1 h in blocking solution with antibodies against FANCD2 (ab2187, Abcam), PhosphoDNA-PKcs (Ser2056 ab18192, Abcam), MDC1 (ab11169, Abcam), γH2AX (JBW301, Upstate), 53BP1 (MAB3802, Millipore), Mre11 (GTX70212, GeneTex), Rad51 (PC130, Calbiochem), RIF1 (A300–569A, Bethyl), RAP80 (NBP1–87156, Novus) and acetylated lysine (MA1–2021, Thermo). Primary antibody detection was achieved by incubation with anti-rabbit or anti-mouse Alexa Fluor 488 or 594 secondary antibodies (Invitrogen) for 30 min at room temperature. Slides were mounted in DAKO mounting medium supplemented with DAPI (Sigma) and examined at a magnification of 63x using a fluorescence microscope (Zeiss Axio Observer Z1) equipped with an ORCA-ER camera (Hamamatsu). The microscope and camera parameters were set for each series of experiments to avoid signal saturation. Image processing and analysis were performed using ImageJ software (http://rsb.info.nih.gov/ij/).

### Cell fractionation

Cell pellets were resuspended in solution A (Hepes pH 7.9 10 mM, MgCl_2_ 1.5 mM, sucrose 12%, glycerol 10%, DTT 1 mM, protease and phosphatase inhibitors) supplemented with 0.1% Triton X100. After 5 min of incubation on ice, the cells were centrifuged for 4 min at 1300 g, and the soluble proteins (S1) were collected. The nuclei were incubated for 10 min on ice in solution B (EDTA 3 mM, EGTA 0.2 mM, DTT 1 mM, protease and phosphatase inhibitors) and then centrifuged for 4 min at 1700 g, and the soluble nuclear proteins (S2) were collected. The chromatin (P2) was mixed in Laemmli 1× buffer and sonicated. The S1 and S2 fractions were mixed with Laemmli 4× buffer. All fractions were denatured by boiling and analyzed by western blotting. Whole-cell extracts (WCE) were obtained with the standard protocol described above from a small portion of the cells that were further used for fractionation.

### Proximity ligase assay (PLA) protein interaction assay

Cell culture and primary antibody staining were performed according to the classical immunofluorescence procedure. The PLA assay (Olink Bioscience) was conducted using the manufacturer's protocol. Dot quantification was achieved automatically using ImageJ software. Negative controls using only a single primary antibody are reported in Supplemental Figure S1D.

## RESULTS

### FA pathway deficiency leads to a non-programmed usage of c-NHEJ in response to mitomycin C treatment

An ongoing replication fork cannot advance through or bypass the obstacle created by a DNA ICL. Consequently, in association with replication fork collapse, the formation of a DSB is the major outcome of an ICL-stalled replication fork. Subsequently, DSB provides a substrate for fork reconstitution and replication rescue, which are mediated by several proteins involved in HR repair (1–3).

**Figure 1.**
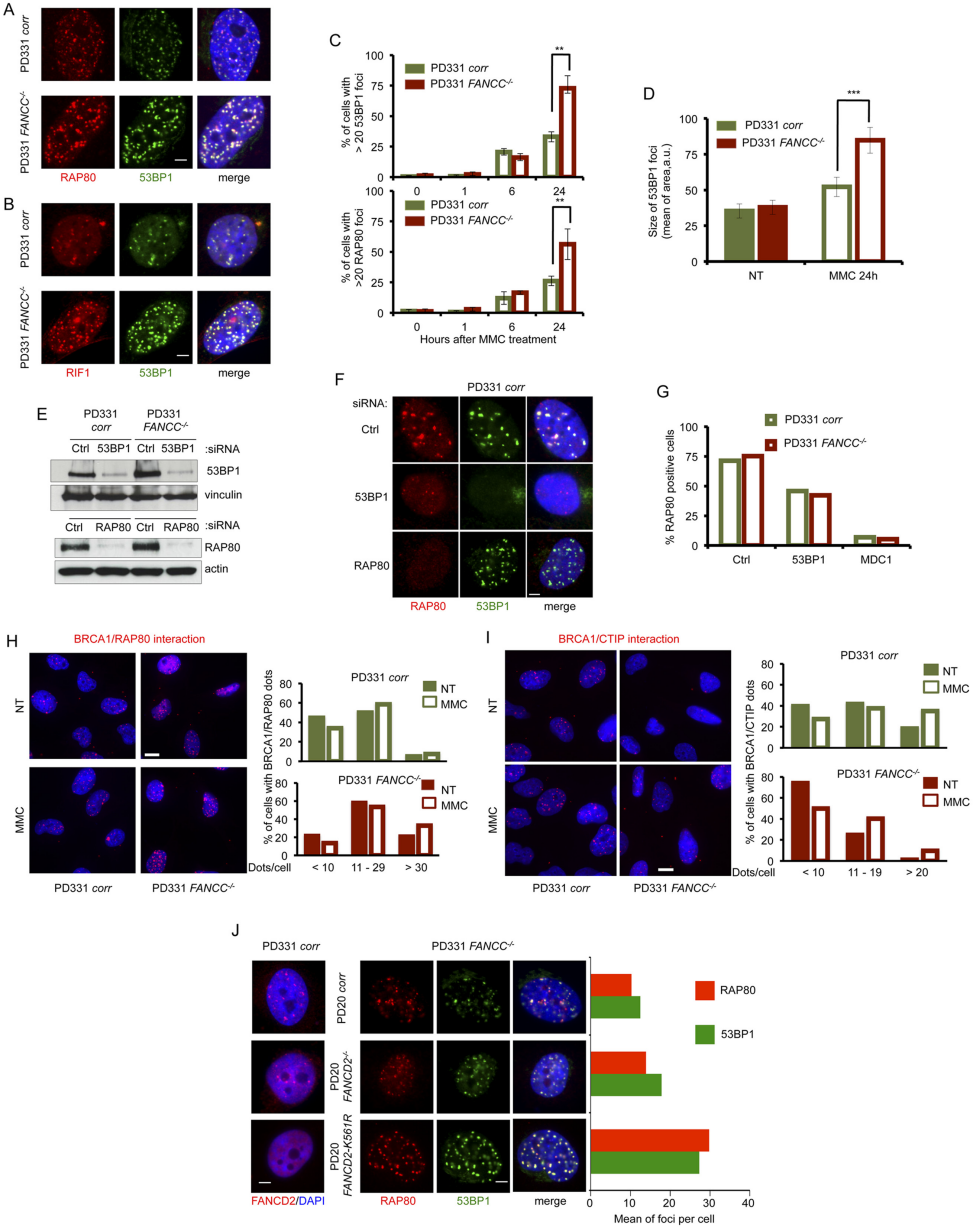
53BP1 and RAP80 altered foci accumulation in FA pathway-deficient cells. (**A**) Representative images of RAP80 (red) and 53BP1 (green) foci in nuclei (DAPI stained, blue) of FANCC-mutated (PD331 *FANCC^−/−^*) and -corrected (PD331*corr*) cells 24 h after MMC exposure (1 μg/ml/1 h). White line: 2 μm. (**B**) Representative images of RIF1 (red) and 53BP1 (green) foci in nuclei (DAPI stained, blue) of FANCC-mutated (PD331 *FANCC^−/−^*) and -corrected (PD331*corr*) cells 24 hours after MMC exposure (1 μg/ml/1 h). White line: 2 μm. (**C**) 53BP1 and RAP80 foci quantificationin untreatyed cells (0 h) and at different time points (1, 6 and 24 h) following MMC exposure (1 μg/ml/1 h). Histograms represent the mean of three independent experiments. Error bars indicate S.D. The data were analyzed by a Student's *t*-test; ** indicates *P* < 0.01. (**D**) Size (arbitrary unit, a.u.) of 53BP1 foci in untreated conditions and 24 h after MMC exposure (1 μg/ml/1 h) in FANCC-mutated (PD331 *FANCC^−/−^*) and -corrected (PD331*corr*) cells. The cell size was determined using the ImageJ software. The values on the histogram represent the mean of three independent experiments; at least 50 cells were scored each time. Error bars indicate S.D. Data were analyzed by a Student's *t*-test; *** indicates *P* < 0.001. (**E**) Western blot showing the efficiency of the siRNA against 53BP1 and RAP80, as observed 72 h after transfection, in corrected (PD331 corr) and FA-C (PD331 FANCC^−/−^) cells. Vinculin was used as a loading control. siCtrl (control) indicates cells transfected with an untargeted siRNA. (**F**) Representative images of RAP80 (red) and 53BP1 (green) foci in nuclei (DAPI stained, blue) of FANCC-corrected cells (PD331 *corr*) after depletion of 53BP1 or RAP80 by siRNA transfection. Cells were treated with MMC (1 μg/ml/1 h) and analyzed 24 h later. White line: 2 μm. siCtrl (control) indicates cells transfected with an untargeted siRNA. (**G**) Quantification of RAP80 foci-positive cells after transfection with an untargeted siRNA (siCtrl, control), si53BP1 or siMDC1 in FANCC-mutated (PD331 *FANCC^−/−^*) and -corrected (PD331*corr*). Cells presenting more than 5 foci were considered positive. Data represent the mean of two independent experiments with similar results. (**H**) Left. Representative images of BRCA1/RAP80 interaction dots detected with the Proximity Ligation Assay in nuclei (DAPI stained, blue) in FANCC-mutated (PD331 *FANCC^−/−^*) and -corrected (PD331*corr*) cells under untreated conditions or 24 h following MMC exposure (1 μg/ml/1 h). White line: 8 μm. Right. BRCA1/RAP80 interaction dots quantification 24 h after MMC exposure (1 μg/ml/1 h). Histograms represent the pooled data from three independent experiments. Quantification was conducted using ImageJ software. (**I**) Left. Representative images of BRCA1/CtIP (left) interaction dots detected with the Proximity Ligation Assay in nuclei (DAPI stained, blue) in FANCC-mutated (PD331 *FANCC^−/−^*) and -corrected (PD331*corr*) cells under untreated conditions or 24 hours following MMC exposure (1 μg/ml/1 h). White line: 8 μm. Right. BRCA1/CtIP interaction dots quantification 24 h after MMC exposure (1 μg/ml/1 h). Histograms represent the pooled data from three independent experiments. Quantification was conducted using ImageJ software. (**J**) Representative images of FANCD2 foci (red), RAP80 (red) or 53BP1 (green) in PD20 *corr* (*FANCD2*-corrected cells), PD20 (*FANCD2^−/-^* cells) and *PD20 FANCD2 K561R* (PD20 cells expressing a K561R, non-ubiquitinable FANCD2) cells. White line: 2 μm. Histograms represent the number of RAP80 (red) and 53BP1 (green) foci in the three different cell lines in the left. Data represent the mean of two independent experiments with similar results.

Accordingly, following exposure to the classical ICL-inducing agent MMC, DNA repair-proficient cells presented an accumulation of γH2AX, MRE11, MDC1, FANCD2 and RAD51 foci, demonstrating the activation of the HR pathway in response to MMC-induced DNA lesions (Supplemental Figure S1A). In contrast, in spite their capability to assemble γH2AX and MDC1 foci, FANCC-deficient cells displayed a strongly impaired recruitment of MRE11, FANCD2 and RAD51 into subnuclear foci in response to MMC (Supplemental Figure S1A). Moreover, in contrast to the DNA repair-proficient cells, *FANCC^−/−^* cells showed a stronger accumulation of 53BP1, RAP80, RIF1 and pDNA-PKcs subnuclear foci, suggesting that NHEJ, and not HR, was used to resolve the DNA DSBs (Figure 1A–C and Supplemental Figure S1A to S1C). It is noteworthy that 53BP1, RAP80 and RIF1 assembled into foci that largely co-localized in both *FANCC*^−/−^ and *FANCC*-corrected cells, indicating that their mobilization is a physiological response of the cell to the MMC-induced DNA damage (Figure 1A and B and Supplemental Figure S1B and S1C). However, approximately 60% of the FA cells contained more than 20 53BP1 or RAP80 foci per cell 24 h after MMC treatment, while <30% of the FA-corrected cells displayed the same effect (Figure 1C). Moreover, the size of the 53BP1 foci in the FANCC-deficient cells was significantly larger than in FANCC-proficient cells (Figure 1D and Supplemental Figure S1C). Qualitatively similar results were obtained in response to lower doses (100 ng/ml/1 h) of MMC as well as to a prolonged exposure (100 ng/ml/24 h) to the drug (data not shown). In conclusion, FANCC loss-of-function in MMC-treated cells leads to an HR defect related to the inappropriate use of the c-NHEJ pathway. These alterations are potentially mediated by the abnormal recruitment of 53BP1 to DSBs associated with the ICL-stalled replication forks.

53BP1 depletion in MMC-treated cells resulted in a clear reduction in the number, size and brightness of RAP80 foci, whereas the siRNA-mediated downregulation of RAP80 had only a minor, if any, effect on 53BP1 localization (Figure 1E–G). Depletion of MDC1, which is known to regulate both 53BP1 and RAP80 recruitment to ionizing radiation-induced DSBs, is associated with an almost total absence of RAP80 foci assembly following exposure to MMC (data not shown and Figure 1G). Thus, it clearly appears that in response to MMC, RAP80 and 53BP1 act downstream of MDC1, with 53BP1 playing a role in the recruitment and/or in the long-term accumulation of RAP80 to ICL-associated DSBs.

BRCA1, which has been recently added to the list of the FANC proteins and whose chromatin binding ability is defective in FA cells (16,29), has been isolated in four complexes that contain common and mutually exclusive partners (30). Notably, BRCA1 associates with either RAP80, which opposes HR, or CtIP, RAD51 or BACH1/FANCJ, which favor HR. By using proximity ligase assay (PLA) technology (Supplemental Figure S1D), we observed that, in both untreated and MMC-treated conditions, the frequency of cells containing an elevated number of BRCA1/RAP80 dots per cell is higher in FANCC^−/−^ cells than in FANCC corrected cells (Figure 1H), whereas the opposite is observed for BRCA1/CtIP dots (Figure 1I). Thus, our data support and extend previous observations describing abnormalities in the HR and NHEJ pathways, as well as in the subnuclear relocalization of BRCA1 in FA.

Similar results were also obtained in FANCA-deficient cells (data not shown), raising the question of whether the observed defect is associated with the FANCcore complex-dependent monoubiquitination of FANCD2. Consequently, we analyzed the behavior of both 53BP1 and RAP80 in FANCcore complex-proficient but FANCD2-mutated cells (PD20) and in their derivatives that constitutively express a wt (PD-20corr) or a non-monoubiquitinable (PD20KR), K561R mutated, FANCD2 protein. Because the PD20 cell line expresses a low level of a mutated but still monoubiquitinable FANCD2, a small number of FANCD2 foci was observed in these cells following MMC exposure (Figure 1J). However, the exogenous expression of wt FANCD2 rescued the FANCD2 foci defect, and the expression of the K561R mutant impeded the residual formation of FANCD2 foci in PD20 cells. Interestingly, the accumulation, size and brightness of 53BP1 and RAP80 foci were inversely proportional to the cell's ability to assemble FANCD2 foci (Figure 1J).

In conclusion, our data suggest that, in the absence of a proficient FA pathway, the rescue of a DSB at an ICL-stalled replication fork is managed by c-NHEJ downstream of 53BP1, which favors the accumulation of the RAP80/BRCA1 complex instead of the CtIP/BRCA1 complex, limiting DNA end resection and allowing NHEJ progression.

### 53BP1 depletion restores the formation MRE11 foci, restrains the accumulation of pDNA-PK foci and alleviates MMC hypersensitivity in FA cells

**Figure 2.**
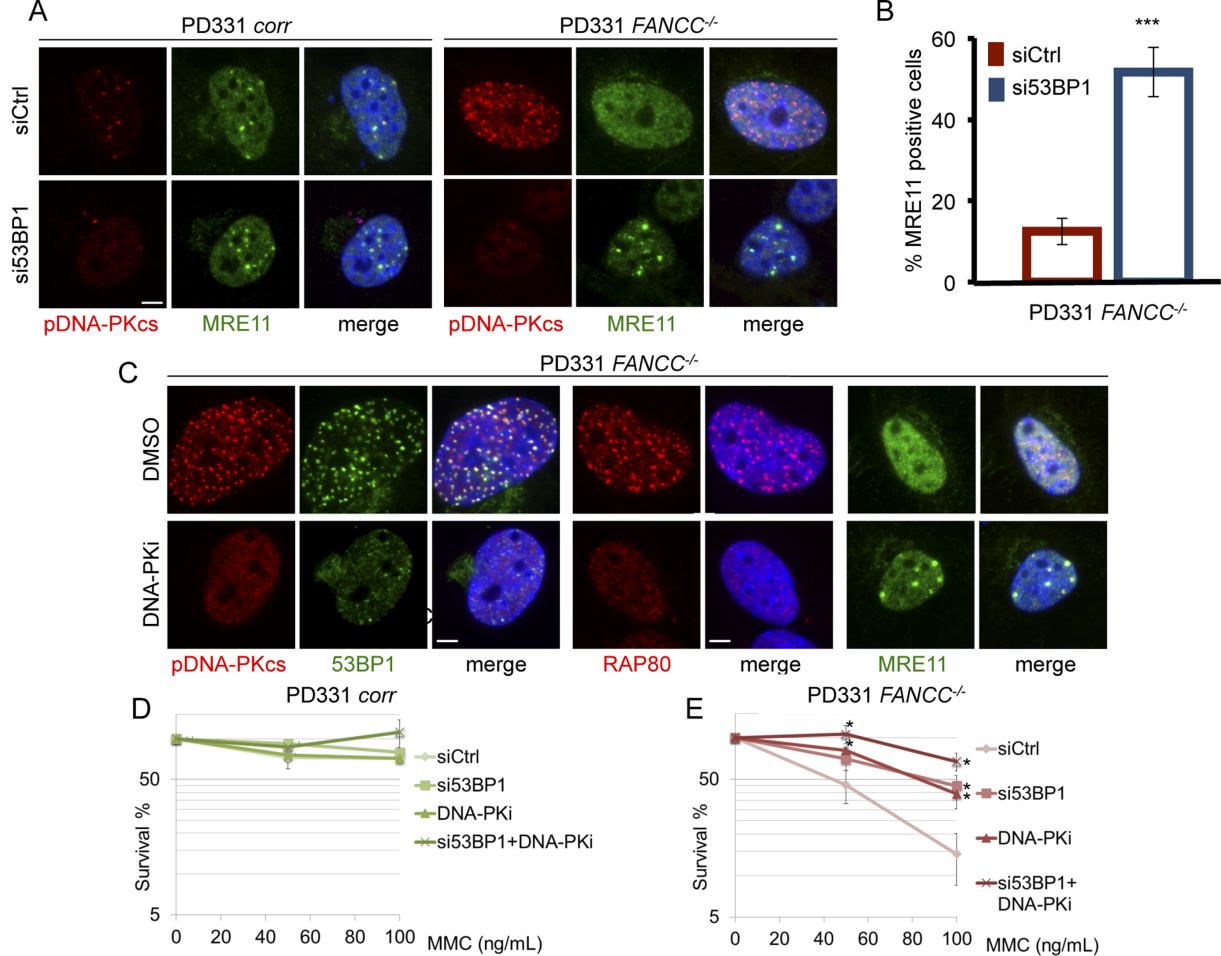
NHEJ pathway inhibition rescue HR and cell survival in FA cells. (**A**) Representative images of pDNA-PKcs (red) and MRE11 (green) foci in nuclei (DAPI stained, blue) of *FANCC*-corrected (PD331 *corr*) or *FANCC*-mutated (PD331 *FANCC^−/−^*) cells in which 53BP1 was depleted by siRNA White line: 2 μm. (**B**) Histogram presents the frequency of MRE11-positive *FANCC^−/−^* cells 48 h after 53BP1 downregulation by siRNA transfection. The presented data are the mean of three independent experiments; error bars indicate S.D. *** indicates *P* < 0.001 using a Student's *t*-test. (**C**) Representative images of pDNA-PKcs (red), 53BP1 (green), RAP80 (red) or MRE11 (green) foci in nuclei (DAPI stained, blue) of FANCC-deficient cells (PD331 *FANC^−/−^*) treated with DMSO or with a DNA-PK specific inhibitor. The cells were treated with DNA-PK inhibitor (DNA-PKi 10 μm) for 2 h before MMC exposure (200 ng/ml). White line: 2 μm. (**D** and **E**) Clonogenic survival of FANCC-proficient (D, PD331 *corr*) or -mutated (E, PD331 *FANCC^−/−^*) cells after 53BP1 depletion by siRNA and/or DNA-PK inhibition. The cells were treated with MMC at the indicated doses. The presented data are the mean of three independent experiments; error bars indicate S.D. * indicates *P* < 0.05 using a Student's *t*-test.

Considering the results described above, we decided to determine the behavior of proteins involved in HR and c-NHEJ in 53BP1-depleted cells in response to MMC. 53BP1 depletion in FA pathway-proficient cells did not alter the formation of MRE11, Ub-FANCD2 and RAD51 foci (Figure 2A and data not shown), but it significantly reduced the formation of pDNA-PKcs foci in MMC-treated FANCC-deficient cells and restored their capability to assemble MRE11 foci (Figure 2A and B). Thus, it appears that as soon as 53BP1 is relieved, MRE11 accumulation may take place in cells where the FA pathway is inactivated. Strikingly, the pharmacological inhibition of DNA-PKcs by exposure to the specific inhibitor NU7441 leads to the loss of 53BP1 and RAP80 foci as well as to the rescue of the MRE11 foci in FANCC-deficient cells (Figure 2C and Supplemental Figure S2A and S2B). This pattern likely suggests that the analyzed processes are highly dynamic and that the abnormal recruitment of 53BP1 in FA cells appears to be the event that causes the accumulation of pDNA-PKcs, failure of which, in turn, is sufficient for HR rescue.

The depletion of 53BP1, the inhibition of DNA-PK or both did not modify the clonogenic survival of FANC-proficient cells upon exposure to MMC (Figure 2D). In sharp contrast, 53BP1 downregulation or DNA-PK inhibition significantly improved the resistance of FA cells to MMC. Indeed, at 100 nM MMC, cell survival increased from 15% to 40% (Figure 2E). More importantly, the concomitant depletion of 53BP1 by treatment with a DNA-PK inhibitor increased the MMC resistance of the FA cells to a level similar to that of corrected cells (Figure 2E).

Consequently, our observations strongly support the hypothesis that FA pathway loss-of-function affects the choice of the DSB repair pathway to be used to repair the broken DNA ends created at stalled replication forks and to rescue replication. Blocking early NHEJ events, such as DSB resection inhibition (performed by 53BP1) and/or the optimal end-tethering (performed by pDNA-PKcs), may switch DSB repair toward HR and revert the MMC hypersensitivity of the FA cells.

### Histone H4 is hypoacetylated in FA cells that fail to recruit TIP60 to damaged chromatin

**Figure 3.**
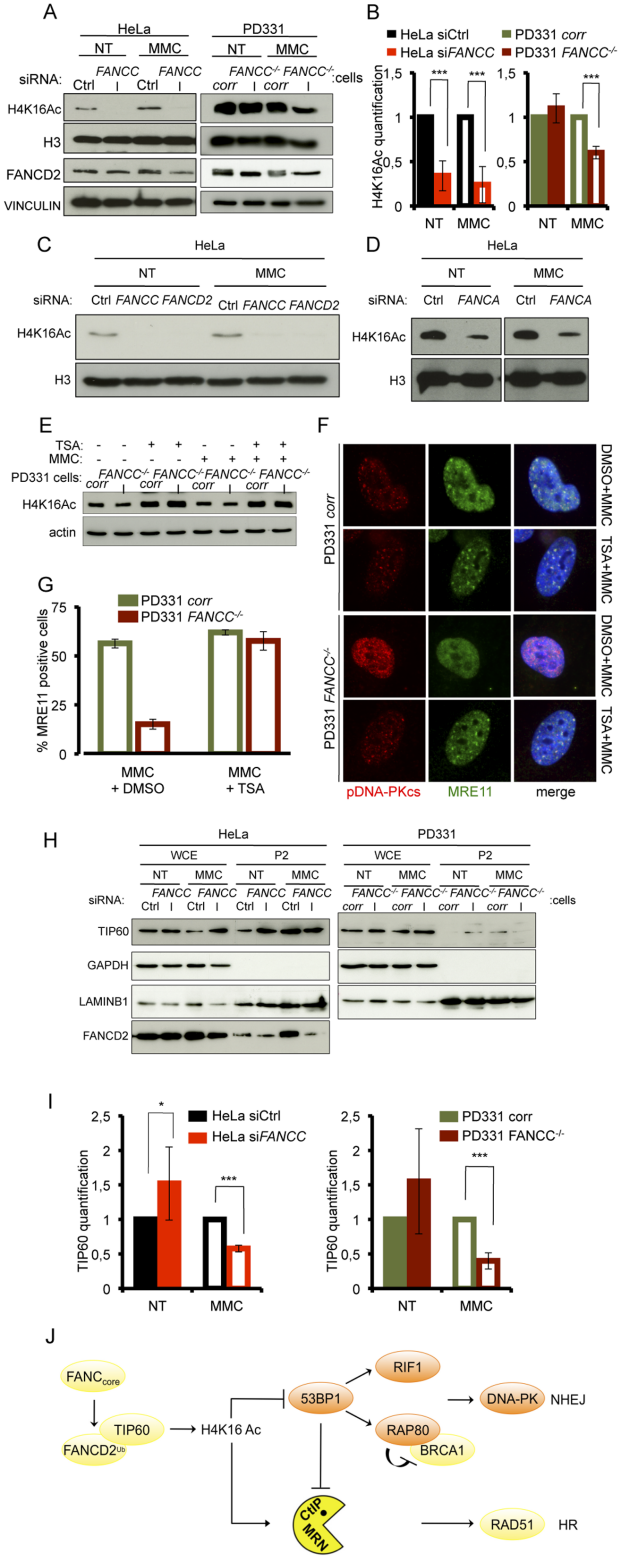
H4K16 acetylation is impaired in MMC-treated FA cells due to altered TIP60 relocalization to damaged chromatin. (**A**) Western blot analysis of H4K16 acetylation following siRNA-mediated FANCC downregulation in HeLa cells (left) and in FANCC-corrected (PD331 *corr*) or -deficient (PD331 *FANCC^−/−^*) cells (right) under untreated conditions (NT) and 24 h after exposure to MMC (1 μg/ml/1 h). FANCD2 is used as control of the loss-of-function of the FANCcore complex activity in absence of FANCC. Vinculin and H3 are presented as loading control. (**B**) Quantitative analysis from four independent experiments of the levels of H4K16 acetylation observed by WB as in (A). In each experiment, H4K16 acetylation was measured by densitometry and normalized relatively to the total H3 levels. Error bars indicate S.D. Statistical analysis: *** indicates *P* < 0.001 using a Student's *t-*test. (**C** and **D**) Western blot analysis of H4K16 acetylation in siFANCC-, siFANCD2- or siFANCA-transfected HeLa cells under untreated conditions (NT) and 24 h after exposure to MMC (1 μg/ml/1 h). H3 is presented as loading control. (**E**) Western blot analysis of the effect of TSA treatment (5 μM) on the levels of H4K16 acetylation in untreated or MMC-treated FANC-corrected (PD331 *corr*) and -deficient (PD331 *FANCC^−/−^*) cells. Actin is presented as loading control. (**F**) Representative images of pDNA-PK (red) and MRE11 (green) foci in nuclei (DAPI stained, blue) of FANCC-corrected (*corr*) or -deficient (*FANCC^−/−^*) cells. The cells were pre-treated with DMSO or TSA (5 μM) for 5 h before MMC (200 ng/ml) exposure and analyzed 24 h later. White line: 2 μm. (**G**) Quantitative analysis of the data presented in (F). Histograms are the mean of three independent experiments. (**H**) Western blot analysis of the subcellular distribution of TIP60 in untreated and MMC-treated FANCC-proficient (Ctrl or *corr*) or -deficient (siFANCC or *FANCC^−/−^*) cells 24 h after exposure to MMC (1 μg/ml/1 h). GAPDH and LaminB1 are used as loading controls. (**I**) Quantitative analysis from four independent experiments of TIP60 relocalization to the chromatin in HeLa cells 48 h after siRNA-mediated FANCC downregulation (left panel) or in FANCC-corrected (PD331*corr*) or deficient (PD331 *FANCC^−/−^*) cells (right panel). Error bars indicate S.D. * indicates *P* < 0.05 and ***P < 0.001 using a Student's *t*-test. (**J**) Simplified model summarizing our data.

After demonstrating that a FANCcore complex or FANCD2 deficiency leads to the inappropriate recruitment of 53BP1 to damaged chromatin, we sought to investigate the mechanism through which the FA pathway promotes the HR pathway for the rescue of stalled forks. 53BP1 attaches to chromatin at DSBs by binding to H4K20me2. Using Western blot analysis, we determined that the levels of H4K20me2 were not affected by FA pathway inactivation. Furthermore, using the PLA approach, we demonstrated that 53BP1 co-localized with H4K20me2 (Supplemental Figure S3A, S3B and S3C). Similar results were also obtained for two other histone post-translational modifications. Indeed, the levels of H3K9me3 and H3K9Ac were also unaffected by the FANCC loss-of-function (Supplemental Figure S3A and S3B). In contrast, Western blot analysis demonstrated that there was a significant decrease in H4K16 acetylation in FANCC-deficient cells, (Figure 3A and B, Supplemental Figure S3A and S3B). Similar results were also observed following FANCA or FANCD2 depletion (Figure 3C and D).

Thus, the FA pathway appears to be required for optimal H4K16 acetylation in response to MMC. Because it has been reported H4K16Ac can conceal H4K20me2 to impede the binding of 53BP1 to damaged chromatin, we sought to verify whether the abnormal NHEJ that occurs in FA cells is the result of defective H4K16 acetylation. Histone acetylation is the finely tuned result of acetyltransferase activities, which deposit acetyl groups at their targets, and deacetylases (HDAC), which remove acetyl groups. Thus, we treated cells with well-characterized HDAC inhibitor trichostatin A (TSA) to increase H4K16 acetylation in response to MMC in both FANC-proficient and FANCC-deficient cells to similar levels (Figure 1E). In line with the general role of H4K16 acetylation in promoting DNA end repair through HR, TSA exposure was sufficient to rescue MRE11 foci formation in FANCC-deficient cells in parallel with a normalization of the pDNA-PKcs signal (Figure 3F and G and Supplemental Figure S3D).

The above observations suggest that the abnormal accumulation of 53BP1 in FANCC-deficient cells is linked to H4K16 hypoacetylation and that the restoration of acetylation by HDAC inhibition partially counteracts FA pathway deficiency by restraining 53BP1 recruitment at DNA damage sites.

It is known that H4K16 is targeted by the acetyltransferase TIP60 (25,31), a FANCD2-interacting protein that acts downstream of FANCD2 during ICL repair (24). Consequently, we suspected that alterations in TIP60 signaling, activity or activation could be the cause of the observed H4K16 hypoacetylation in FANC-deficient cells. As we were unable to identify TIP60 foci in MMC-treated cells using antibodies raised against TIP60 or in cells overexpressing an HA-tagged TIP60, we analyzed the recruitment of TIP60 to the chromatin in MMC-treated cells by Western blotting fractionated cellular extracts. We separated cytoplasmic (S1), soluble nuclear (S2) (Supplemental Figure S3E and S3F) and chromatin-bound proteins (P2) (Figure 3H) in FANCC-proficient and FANCC-deficient cells. We observed a significant increase of the level of TIP60 in the P2 fraction isolated from FANCC-proficient cells treated with MMC (Figure 3H and Supplemental Figure S3G). This finding validates the previously proposed role of TIP60 in ICL repair (24). On the contrary, in FA pathway-deficient cells, we did not detect an increase, but instead a decrease, in the levels of TIP60 bound to chromatin in response to MMC (Figure 3H and Supplemental Figure S3G, S3H and S3I). Indeed, compared to their proficient counterpart, the chromatin level of TIP60 in MMC-treated FANCC-deficient cells is significantly lower (Figure 3I). Moreover, it appears that FANCC-deficiency is associated to a slight increase in TIP60 at the chromatin (Figure 3H and I) in absence of genotoxic stress. This could be due to the redistribution of Tip60 to other partners or to a reduced mobility of non-ubiquitinated FANCD2, which is always associated to the chromatin.

Thus, cells that are unable to monoubiquitinate and relocalize FANCD2 into chromatin-associated subnuclear foci are also unable to optimally load TIP60 at the chromatin in response to MMC exposure. These results suggest that TIP60 is involved in ICL repair through FANCD2-dependent binding to damaged chromatin to trigger H4K16 histone acetylation, an event known to limit 53BP1 recruitment to its docking site H4K20me2 (Figure 3J).

## DISCUSSION

Here, we report that one key function of the FANCcore complex-FANCD2 axis is to promote H4K16 acetylation by TIP60 at the right place and time, which is thought to conceal the contiguous dimethylated K20 residue (25,32) from 53BP1 (33,34). The 53BP1 that is recruited to broken DNA is likely to impede DNA resection and HR, favoring DNA repair through NHEJ (26).

The interaction between TIP60 and FANCD2, which exists constitutively and independently of FANCD2 monoubiquitination, has been previously reported, and both proteins appear to act on a common pathway to rescue ICL-damaged cells (23,24). We determined the possible biological significance of their biochemical and functional interactions. Indeed, we demonstrated that both accumulation of TIP60 at ICL-damaged chromatin and H4K16 acetylation are significantly and specifically reduced in FANCA-, FANCC- and FANCD2-deficient cells. We argue that through its interaction with FANCD2, TIP60 is relocalized onto the damaged chromatin by Ub-FANCD2 to locally acetylate the K16 residue on histone H4, masking the accessibility of dimethylated K20 to 53BP1 and allowing the initiation of the DNA resection. Thus, the genetic instability in FA cells may largely rely on the misregulation of TIP60 loading on the chromatin, which causes 53BP1 accumulation and triggers inappropriate NHEJ. It has been recently demonstrated that FANCD2 interacts with and relocalizes CtIP to damaged DNA in response to ICL (35). This finding suggests that monoubiquitinated FANCD2 regulates the selection of HR to resolve DSBs associated with stalled replication forks at the correct place and time through at least two proteins whose activities favor HR and impede NHEJ: CtIP and TIP60.

Grompe and colleagues, who generated Fancd2/DNA-PKcs double mutant mice, found that the double-mutant cells are equally sensitive to ICLs as the Fancd2-single mutant (20). More recently, it has been shown that FANCD2^−/−^53BP1^−/−^ and FANCD2^−/−^Ku70^−/−^ mouse embryonic fibroblasts (MEFs) are more sensitive to ICLs than FANCD2^−/−^ MEFs, suggesting that FANCD2 function cannot be rescued by NHEJ depletion/inactivation (36). In contrast, Adamo et al. reported that exposure of FANCA^−/−^ or FANCC^−/−^ MEFs to a DNA-PK inhibitor partially restored their sensitivity to MMC (21). Moreover, these authors reported that DNA-PK inhibitors largely rescue the ICL-induced sensitivity of FANCA- or FANCD2-siRNA-depleted HeLa cells and that the siRNA-mediated depletion of NHEJ protein KU80 partially recovers the MMC hypersensitivity of human fibroblasts belonging to the FA-C and FA-D2 complementation groups. The discordant data may reflect the fact that either the function of FANC proteins or the mechanisms that influence the choice between HR and NHEJ may not completely overlap between mice and humans. Further in-depth analyses are required to address this point.

In conclusion, our findings validate and extend previously published observations and provide a mechanistic explanation for why and how the c-NHEJ proteins 53BP1, RIF1, RAP80 and pDNA-PK accumulate at damaged chromatin instead of MRE11, CtIP and RAD51 in FA pathway-deficient cells. We propose that in addition to promoting replication fork stabilization, ICL incisions and TLS, the FA pathway drives ICL-induced DSB repair through HR by restricting 53BP1 accumulation and enabling DNA resection by mediating TIP60 activity on the chromatin. Consequently, in FA cells, the c-NHEJ pathway is largely responsible for the cellular and chromosomal hypersensitivity to DNA damage. Accordingly, the siRNA-mediated depletion of 53BP1, DNA-PK or HDAC inhibition reduce the activation of the c-NHEJ pathway, promoting the rescue of stalled forks by HR and increasing the resistance of FA cells to MMC. Finally, our data provide the first evidence for the involvement of the FA pathway in chromatin modification, opening new avenues of investigation.

## Supplementary Material

SUPPLEMENTARY DATA
