# Supplementary material for: Impaired TIP60-mediated H4K16 acetylation accounts for the aberrant chromatin accumulation of 53BP1 and RAP80 in Fanconi anemia pathway-deficient cells
Source: Nucleic Acids Res. 2015 Oct 7;44(2):648–56. doi: 10.1093/nar/gkv1019 (PMC4737135; doi:10.1093/nar/gkv1019)
Supplement: SUPPLEMENTARY DATA [file supp_44_2_648__index.html]

Impaired TIP60-mediated H4K16 acetylation accounts for the aberrant chromatin accumulation of 53BP1 and RAP80 in Fanconi anemia pathway-deficient cells — Impaired TIP60-mediated H4K16 acetylation accounts for the aberrant chromatin accumulation of 53BP1 and RAP80 in Fanconi anemia pathway-deficient cells — SUPPLEMENTARY DATA 

# Impaired TIP60-mediated H4K16 acetylation accounts for the aberrant chromatin accumulation of 53BP1 and RAP80 in Fanconi anemia pathway-deficient cells

## SUPPLEMENTARY DATA

- SUPPLEMENTARY DATA
