# Supplementary material for: Impaired TIP60-mediated H4K16 acetylation accounts for the aberrant chromatin accumulation of 53BP1 and RAP80 in Fanconi anemia pathway-deficient cells
Source: Nucleic Acids Res. 2015 Oct 7;44(2):648–56. doi: 10.1093/nar/gkv1019 (PMC4737135; doi:10.1093/nar/gkv1019)
Supplement: SUPPLEMENTARY DATA [file supp_gkv1019_nar-01711-x-2015-File005.pdf]

Impaired TIP60-mediated H4K16 acetylation restrains homologous recombination in Fanconi anemia pathway-deficient cells.

Emilie RENAUD[1,2,3], Aurelia BARASCU[1,2,3], and Filippo ROSSELLI[1,2,3]

(1) Université Paris Sud, Laboratoire «Stabilité Génétique et Oncogenèse», Equipe Labellisée La Ligue Contre Le Cancer.
(2) CNRS - UMR 8200.
(3) Institut de Cancérologie Gustave-Roussy.

SUPPLEMENTAL FIGURES LEGENDS

SUPPLEMENTAL FIGURE LEGENDS

Supplemental Figure 1:

A) Mitomycin C-induced formation of foci containing proteins involved in homologous recombination or non-homologous end joining in FANCC-deficient (PD331 *FANCC*$^{-/-}$) and proficient (PD331 *corr*) cells.

Representative images of FANCD2 (red), MRE11 (green), Rad51 (Green), MDC1 (red), γH2AX (green) pDNA-PKcs (red) 53BP1 (green) and 53BP1 (red) recruitment in FANCC-deficient (PD331) and -corrected (PD331corr) cells 24 hours after MMC treatment (1 µg/mL/1 h). The nuclei were stained with DAPI (bleu). White line: 2 µm.

B: Mitomycin C-induced RAP80 (red) and 53BP1 (green) foci in FANCC-deficient (PD331 *FANCC*$^{-/-}$) and proficient (PD331 *corr*) cells. Examples of larger images showing foci in several cells.

C: Representative images of the level and size of 53BP1 foci (red) 6 hours and 24 hours following mitomycin C exposure in FANCC-deficient (PD331 *FANCC*$^{-/-}$) and proficient (PD331 *corr*) cells.

D: Negative controls for the Proximity Ligation Assay.

The PLA protocol was performed with a single primary antibody (BRCA1, RAP80 or CTiP) in MMC-treated corrected cells or with two primary antibodies (BRCA1/RAP80 or BRCA1/CTiP). White line: 6 µm. Right panel: Quantitative analysis of the data represented in left. The number of dots/cell was measured automatically using ImageJ software.

Supplemental Figure 2:

Effect of DNA-PK inhibition on 53BP1 (**A**, green) and RAP80 (**B**, red) on mitomycin C-induced foci formation in FANCC-deficient (PD331 *FANCC*$^{-/-}$) cells .

Supplemental Figure 3:

A: Western blot analysis of the indicated histone modifications in PD331 FANCC-deficient (PD331 *FANCC*$^{-/-}$) and proficient (PD331 *corr*) cells in untreated conditions and 6 hours or 24 hours after exposure to MMC (1 *µ*g/mL/1 h).

B: Quantitative analysis from three independent experiments of the indicated histone

post-translational modifications. Error bars indicate S.D. Statistical analysis: ** indicates $p < 0.01$ using a Student T test.

C: PLA analysis showing the colocalization and proximity of 53BP1 and H4K20Me2 in mitomycin C-treated in proficient (PD331 *corr*) and FANCC-deficient (PD331 $FANCC^{-/-}$) cells.

D: Images showing the effect of TSA treatment on p-DNA-PKcs (red) and MRE11 (green) foci in mitomycin C-treated PD331 $FANCC^{-/-}$ cells.

E and F: S1 and S2 fractions of the same cellular extracts presented in Figure 3H.

G and H: Altered localization of TIP60 in the P2 fraction as a consequence of siRNA-mediated depletion of FANCC, FANCD2 or FANCA in HeLa cells 24 hours after treatment with MMC and in untreated conditions (NT).

I: TIP60 relocalization to the chromatin in FANCD2-corrected vs K561R-expressing FANCD2-deficient cells under untreated conditions (NT) or in response to MMC exposure.

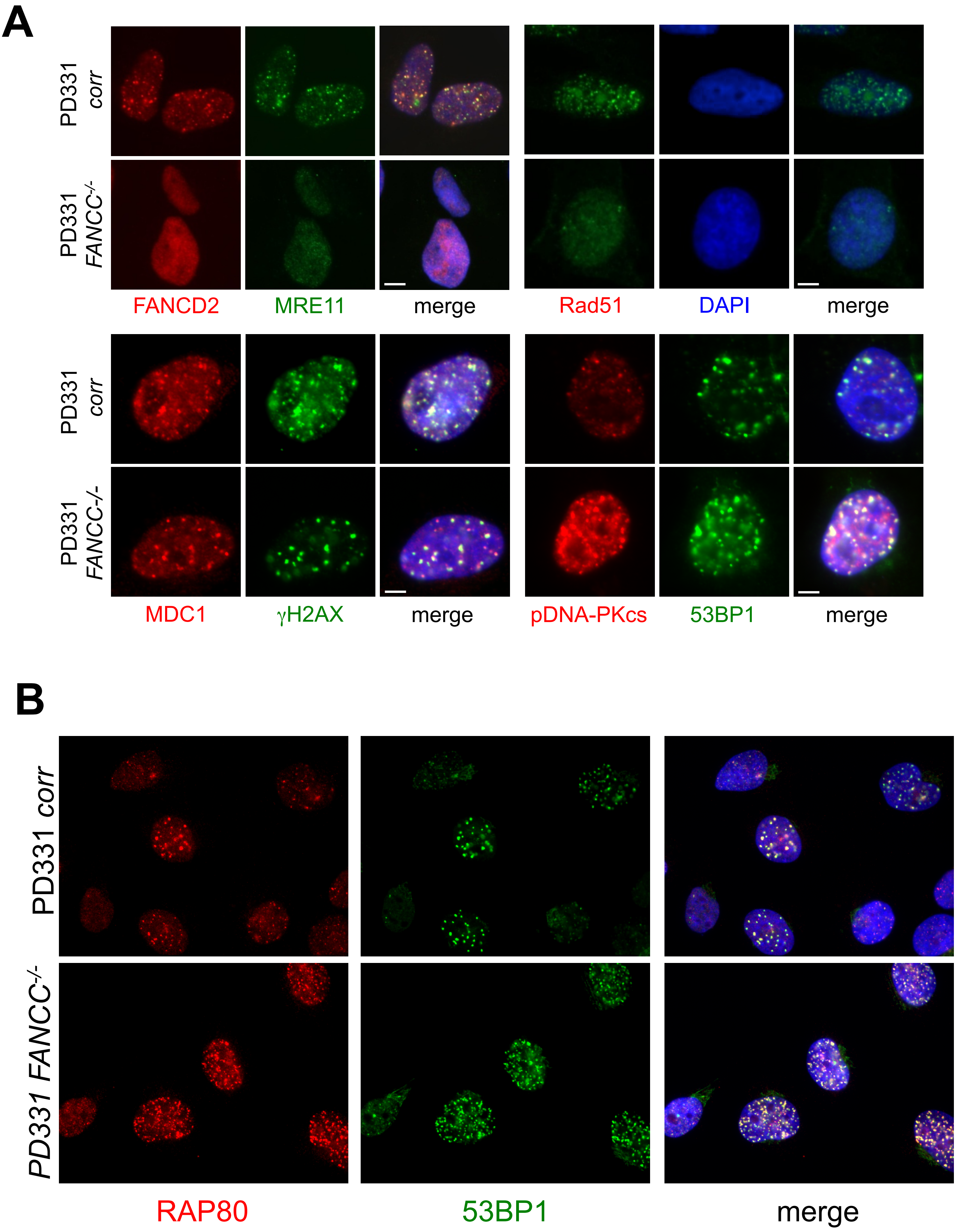

Supplemental Figure 1 (part A) – Renaud et al.

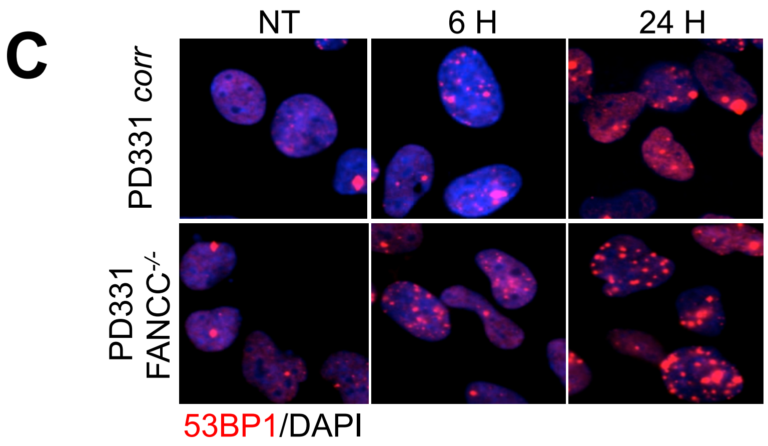

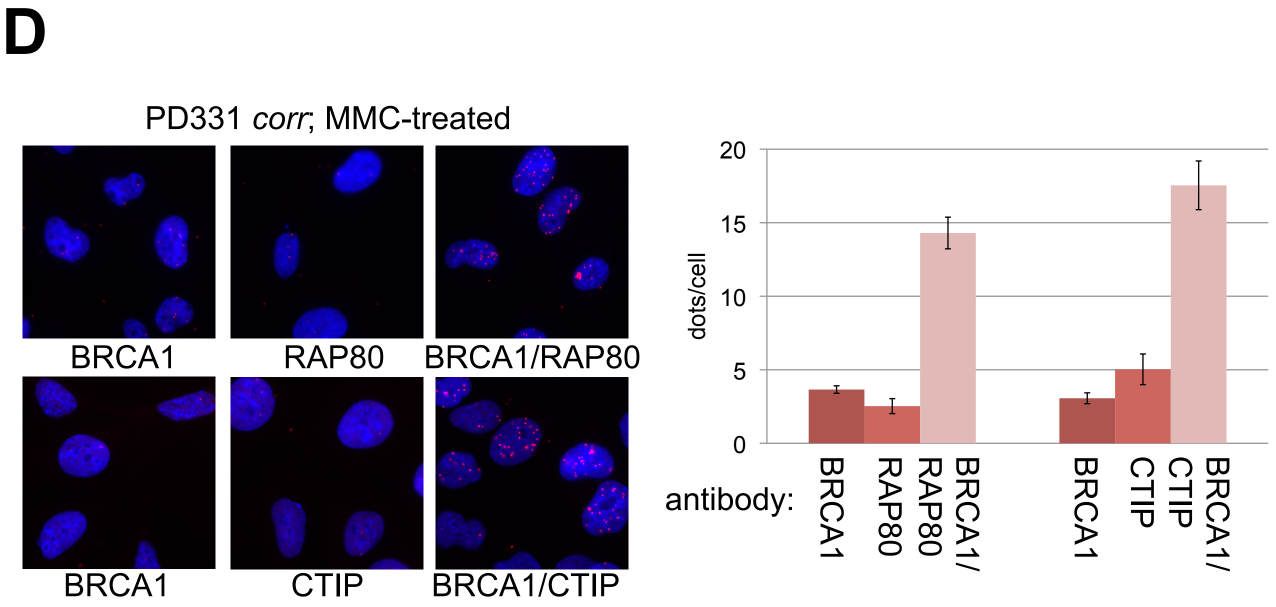

Supplemental Figure 1 (part B) – Renaud et al.

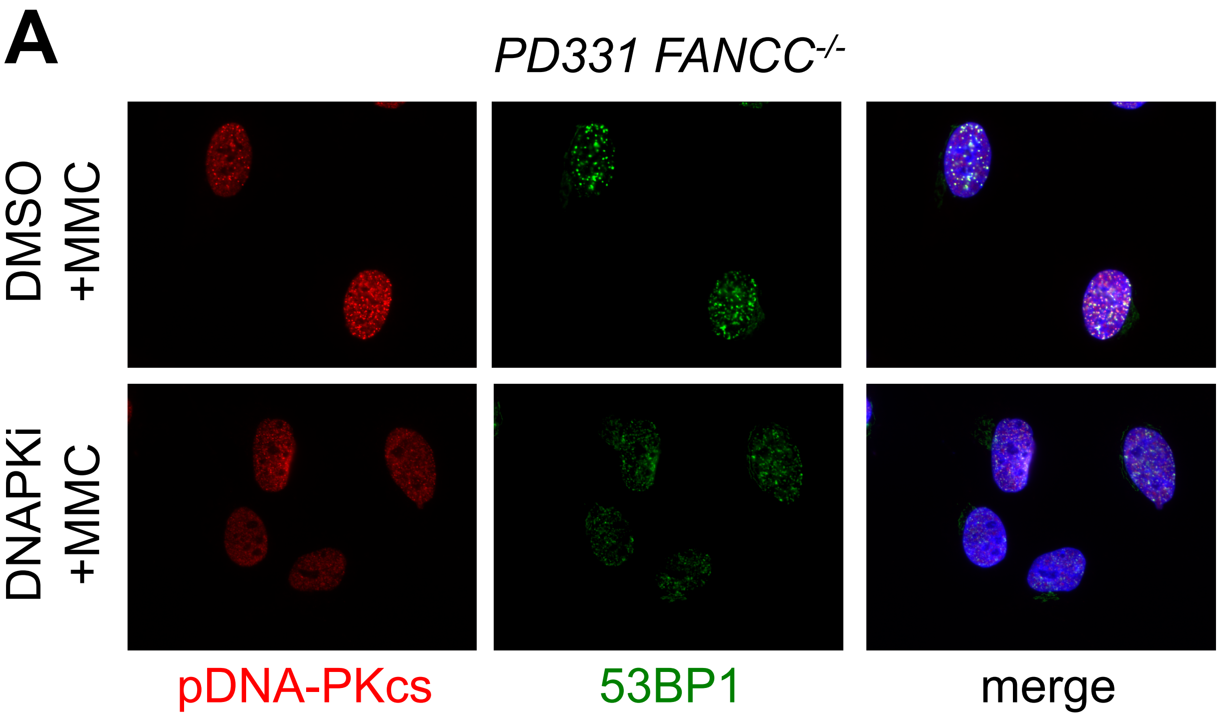

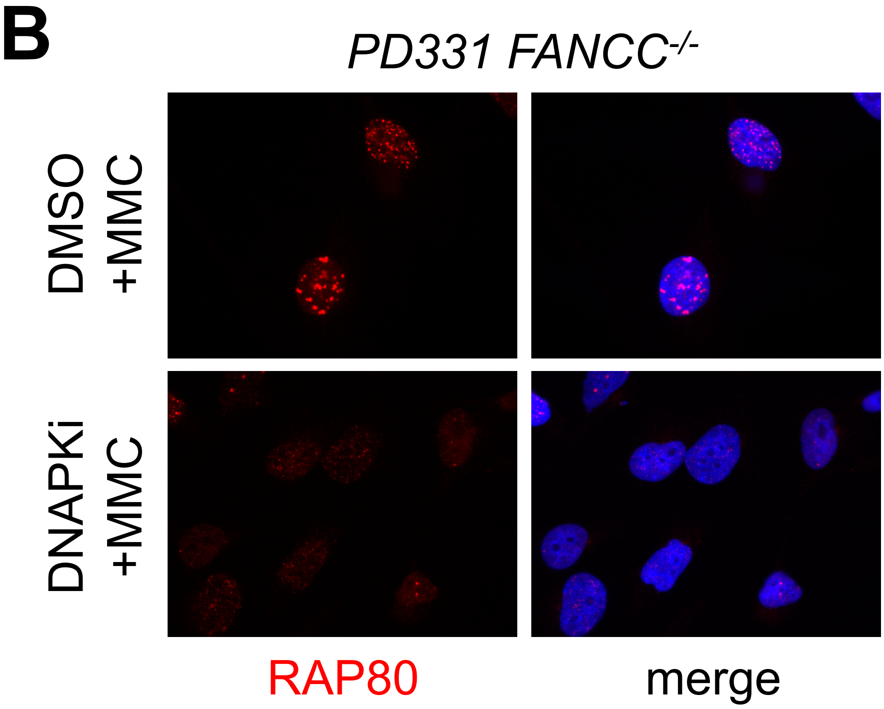

Supplemental Figure 2 – Renaud et al.

Supplemental Figure 3 Part A – Renaud et al.

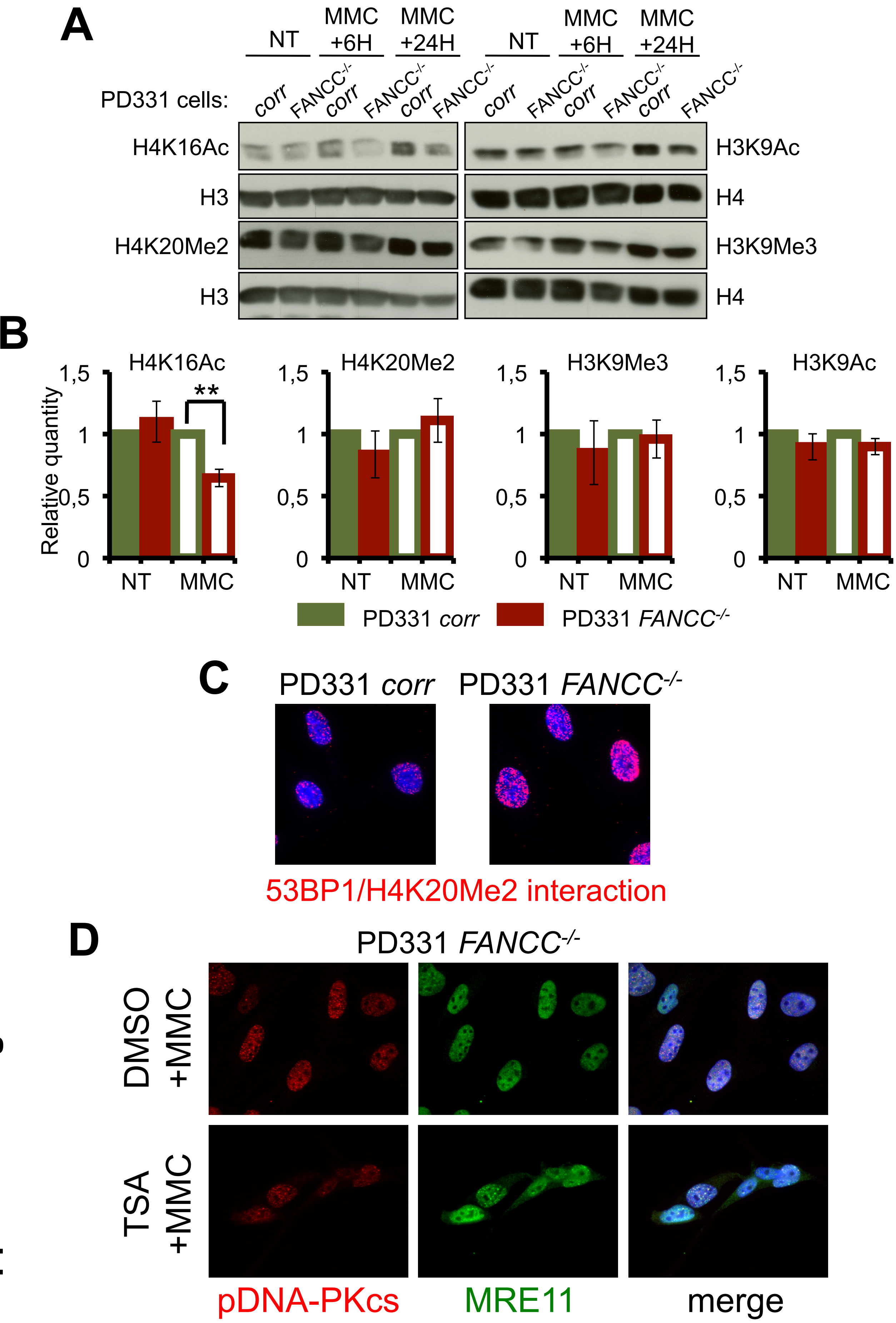

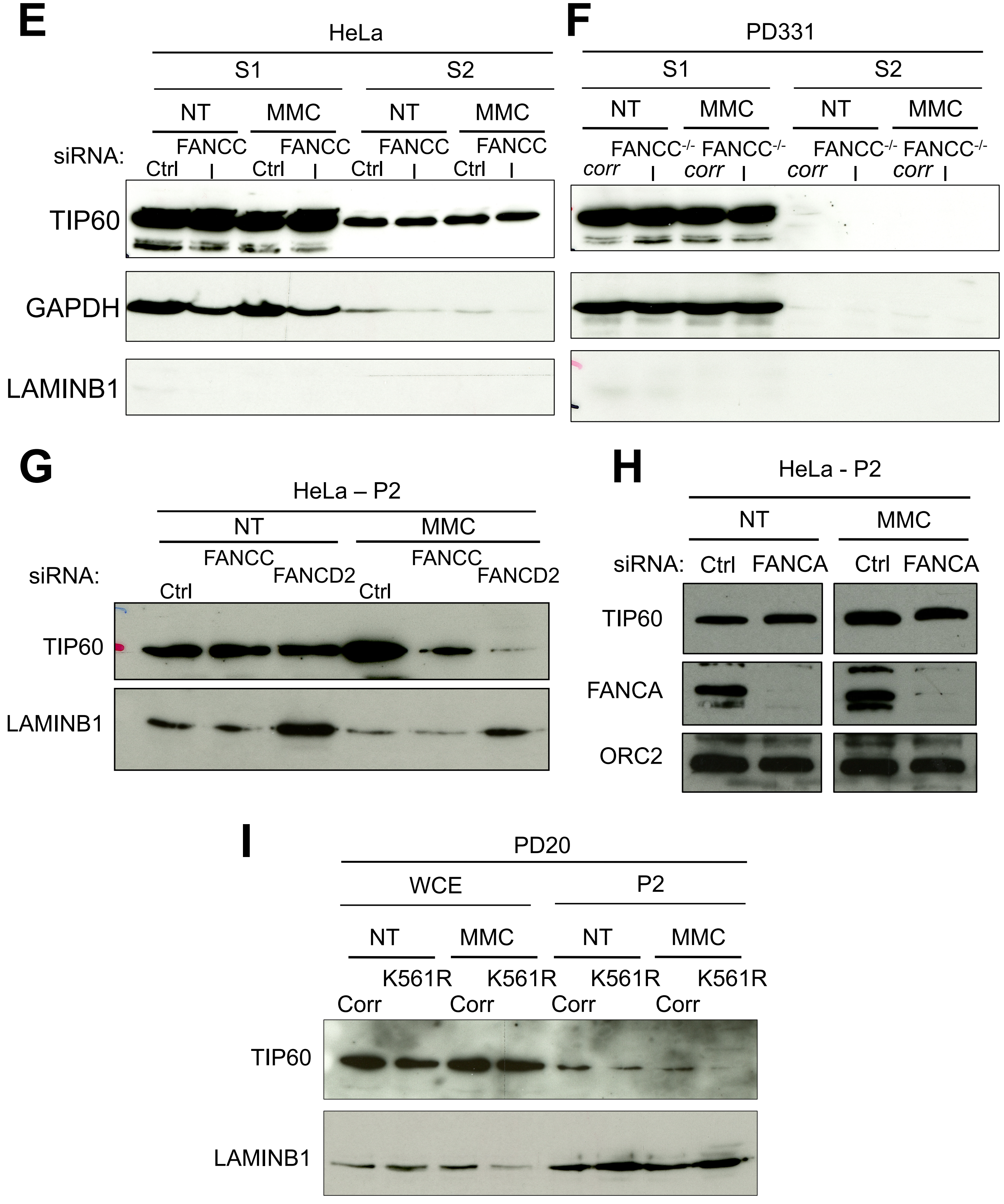

Supplemental Figure 3 Part B – Renaud et al.
